# Exploring Attachment in Adults With Autism Spectrum Disorder: A Systematic Review

**DOI:** 10.62641/aep.v53i4.1928

**Published:** 2025-08-05

**Authors:** Alexandra Sonfelianu, Francisco González-Sala, Laura Lacomba-Trejo

**Affiliations:** ^1^Faculty of Psychology and Speech Therapy, Universitat de València, 46010 València, Spain; ^2^Developmental and Educational Psychology Department, Faculty of Psychology and Speech Therapy, Universitat de València, 46010 València, Spain

**Keywords:** autism spectrum disorder, attachment, adults, systematic review

## Abstract

**Background:**

Attachment styles may exert an influence on emotional regulation, specifically, secure attachment has been associated with more adaptative emotion regulation strategies and enhanced adult well-being. Despite the recognized importance of secure attachment in promoting mental health and well-being, little is known about how attachment styles manifest and evolve in adults with autism spectrum disorder (ASD). This systematic review examines *“What is the connection between attachment styles and psychological, relational, and clinical variables in individuals with ASD or autistic traits, according to scientific research?”*.

**Methods:**

A literature search, adhering to Preferred Reporting Items for Systematic Reviews and Meta-Analyses (PRISMA) guidelines, was performed across PubMed, Web of Science and ProQuest Central databases. Using HubMeta, 12 studies were selected based on set criteria. Two independent reviewers conducted the entire process, from searching and selecting studies to extracting data and assessing quality. Inter-rater agreement was high, with kappa values ranging from 0.94 to 1.

**Results:**

The sample included 91,078 (98.99%) women and 346 (0.38%) men. Studies assessed attachment, ASD or autistic traits (Broader Autism Phenotype, BAP), intelligence quotient (IQ), depression, anxiety, stress, marital and relationship satisfaction, emotional availability and intelligence, empathy, ASD difficulties, personality traits and motivational processes, and gaming disorder. Individuals with ASD show higher rates of insecure attachment than general population and this could have an impact on their mental health and well-being. Parents with ASD also show difficulties when establishing the bond with their children.

**Conclusion:**

This review highlights the importance of developing interventions with adults with ASD with the aim to establish better bonding and reaching greater well-being and mental health.

**The PROSPERO Registration:**

CRD42024628086, (https://www.crd.york.ac.uk/prospero/display_record.php?ID=CRD42024628086).

## Introduction

According to Bowlby [1, 2], attachment is an inherent human need to stay close to significant caregivers, especially when feeling anxious or threatened. This fundamental drive is biologically rooted and serves to ensure child’s safety, survival, and overall well-being [3, 4], emphasizing its crucial role in healthy social and emotional development [5]. Bowlby [1] and Ainsworth *et al*. [6] also highlighted the importance of attachment in protecting the child, noting that secure attachment fosters emotional resilience and healthy relationships, whereas insecure attachment can negatively affect social and emotional well-being [7].

This essential bond forms gradually through consistent and nurturing interactions between the child and their caregiver during the early years of life [8], which shapes a child’s understanding of relationships [9], and are heavily influenced by the caregiver’s emotional availability and responsiveness to the child’s needs [1, 10, 11]. A failure to establish a secure bond with the primary caregiver can result in lasting feelings of insecurity, fear, and mistrust [4].

As children grow, their attachment behaviors evolve from physical closeness in infancy [1] to seeking emotional support in later childhood [12, 13], with early caregiver bonds crucial for lifelong social development as children mature [14]. Once the bond is established, a child’s attachment tends to remain relatively stable over time [1], though it can be influenced by significant life experiences that impact their relationship attachments [15]. These early attachment experiences significantly impact a child’s development, shaping their personality, mental health and relationships [16]. Thus, secure attachment promotes resilience while insecure attachment can negatively affect well-being [7].

Regarding attachment styles, building upon Bowlby’s work, Mary Ainsworth identified three primary attachment styles: (a) secure, (b) insecure-resistant, and (c) insecure avoidant [17]. More recently, a fourth attachment style, disorganized/disoriented, has been identified [18]. While these styles are often viewed as distinct categories, Brennan *et al*. [19] proposed a dimensional approach to understanding adult attachment. They identified two key dimensions: avoidance and anxiety. Individuals with avoidant attachment struggle to trust and rely on others, while those with anxious attachment often fear abandonment and seek constant closeness. Secure attachment, in contrast, is characterized by low levels of both avoidance and anxiety [20].

Given the fundamental role of attachment in shaping social and emotional development, it is particularly relevant to explore how attachment functions in populations with distinct developmental challenges, such as individuals with autism spectrum disorder (ASD). The importance of studying attachment styles in ASD stems from earlier beliefs that children with ASD, due to their difficulties in social interactions and communication, are incapable of forming secure attachments [21]. However, more recent studies have shown that individuals with neurodevelopmental disorders [such as attention-deficit/hyperactivity disorder (ADHD), ASD, dyslexia, language disorders, etc.] can indeed form secure attachments, but individuals with ASD show more challenges at doing so compared to their neurotypical peers [14]. Thereby, ASD is a neurodevelopmental disorder defined by lasting difficulties in social communication and interaction, combined with restricted, repetitive patterns of behavior, interests, and/or activities [22, 23]. The number of ASD diagnostic has grown [24], with a prevalence of 0.60% [25]. A similar increase of diagnoses has been observed in Spain, with a prevalence of 1.94 [26]. Also, ASD is more prevalent in boys than in girls, with a male-to-female ratio of approximately 4.3:1 [27]. However, recognizing ASD in girls can be challenging, which may contribute to an underestimation of its prevalence in the female population [28].

In light of the growing number of ASD diagnoses, researchers have increasingly focused on identifying subtle autistic-like traits in the general population, even in nonclinical population, and refers to this collection of traits as the broader autism phenotype (BAP) [29], which is characterized of three key elements: aloof personality, pragmatic language deficits, and rigid personality [30]. Despite being a relatively recent concept, BAP is supported by a growing body of research which suggests that a milder, subclinical form of ASD with a genetic basis exists within the general population [31].

Despite some theories linking ASD to attachment issues [32], children with ASD can form secure attachments [14]; however, ASD severity, co-occurring conditions, and age can affect attachment quality [5]. Risk factors for insecure attachment in children with ASD include parental stress, the severity of ASD symptoms, co-occurring developmental disorders, and poor social skills and family mental health issues [33].

Studying attachment in children with ASD presents significant challenges due to the complex interplay between their social communication difficulties and their attachment relationships [33, 34]. Despite these challenges, research has reported that children with ASD engage in attachment behaviors, such as seeking proximity to caregivers and showing a preference for their company over strangers [14]. However, the developmental trajectory of attachment in children with ASD may differ from that of typically developing children [5]. While there is substantial knowledge about attachment in children with ASD, despite the recognized importance of secure attachment in promoting mental health and well-being [35] and the established link between adult attachment styles and psychological adjustment in the general population [13, 36, 37], little is known about how attachment styles manifest and evolve in adults with this disorder [12] as most research on attachment in ASD has focused on younger children [5, 38].

To address this gap in our understanding, this review systematically examines the existing literature on attachment styles in adults with ASD with the aim of clarifying what is already known, identify patterns and propose future directions. The recognized importance of secure attachment emphasizes the need for further research in this area [5] as it has been seen that attachment style may exert an influence on emotional regulation, specifically, secure attachment has been associated with more adaptative emotion regulation strategies [39] and enhanced adult well-being [40].

This review seeks to broaden understanding in this field by systematically examining attachment styles in adults with ASD, providing insights into their developmental trajectories and psychological implications. This review is guided by the following research question, formulated using the following PECO (Population = individuals with ASD or autistic traits, Exposure = Attachment style, Comparison = not applicable, Outcome = psychological, relational, and clinical variables) strategy [41]: *“What is the connection between attachment styles and psychological, relational, and clinical variables in individuals with ASD or autistic traits, according to scientific research?”*.

## Methods

### Search Strategy

The current study has been conducted following the guidelines proposed by the Preferred Reporting Items for Systematic Reviews and Meta-Analyses (PRISMA) statement [42]. Two independent researchers (F.G-S and A.S) conducted literature searches across three major databases: ProQuest (18th December 2024), PubMed (18th December 2024) and Web of Science (13th December 2024). The search strategy incorporated key concepts, employing Boolean expressions (using MeSH terms) in the databases: (autis* OR autism* OR ASD OR ASC OR autistic traits OR Asperger) AND (attachment OR bond*). The final literature search was conducted on December 18, 2024, to ensure the inclusion of the most recent studies. The databases consulted provide records dating back to 1943. The present Systematic Review has been registered with PROSPERO under the registration number CRD42024628086.

### Eligibility Criteria

The following inclusion criteria were applied to all articles considered for this study: (a) Spanish and English articles; (b) no temporal criterion due to the limited available literature; (c) adults with a diagnosis of ASD or with autistic traits (BAP); (d) reference attachment and autism or BAP; and (e) include comparative studies, controlled clinical trials, observational studies or preprints.

Articles were excluded if they addressed the following: (a) topics not focused on attachment and autism in adults; (b) systematic reviews, narrative reviews, case studies or descriptive articles; and (c) other types of documents such as books, interviews, lectures, chapters, grey literature, dissertations and conferences or congresses.

### Procedure

**Fig. 1.**
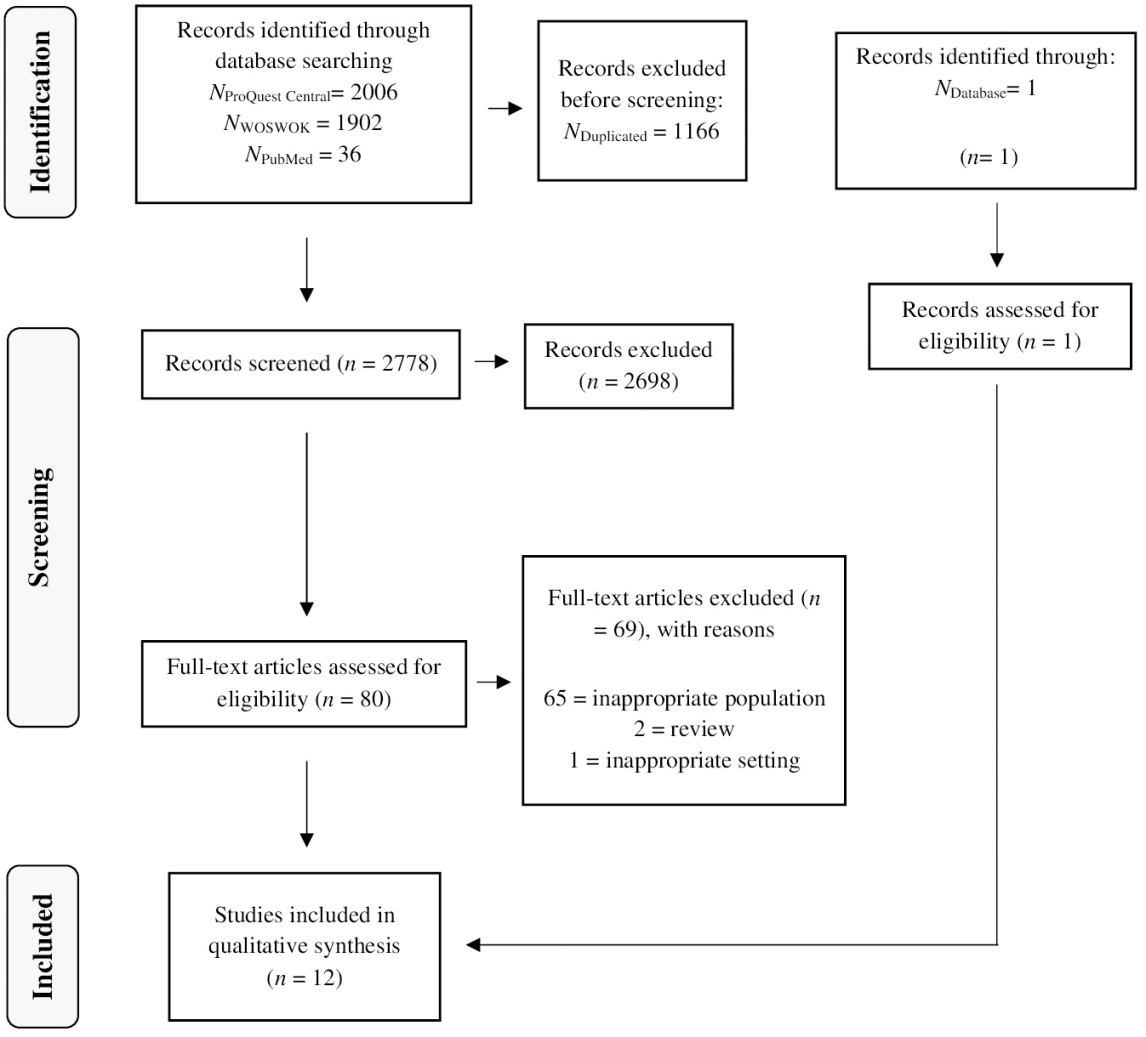
**Flowchart of the selection process**.

To manage the research process, all records were systematically uploaded to HubMeta [43], a web-based data entry system. Duplicate articles were removed automatically. Subsequently, based on a screening of titles and abstracts, articles that did not satisfy the inclusion criteria were discarded. Articles selected by any of the two reviewers (F.G-S and A.S-S), or those where there was disagreement between their independent assessments, were subjected to a further blinded review to ensure they met the established inclusion and exclusion criteria. Disagreements regarding article selection were resolved by discussion. Of the 3944 articles initially examined, only 12 were ultimately included in the final study after removing duplicates and excluding articles that did not meet specific criteria, such as study type and relevance. A visual representation of the entire selection process is provided in Fig. 1.

Descriptive statistics were used to summarise the characteristics of the included studies. Percentages and means were calculated to provide an overview of the sample distributions. Furthermore, to evaluates judge agreement, Cohen’s Kappa index (κ) was used. According to established guidelines [44, 45], values between –1 and 0.40 indicate unsatisfactory agreement, 0.41 to 0.75 are satisfactory, and 0.76 or higher represent excellent agreement. In both the first (κ = 0.94) and second (κ = 1) selection phases, agreement among judges was excellent. However, due to the significant heterogeneity of the results, particularly regarding the variables investigated and the assessment tools employed, a subsequent meta-analysis was deemed inappropriate.

### Quality Assessment

The quality of the selected articles was assessed by A.S, employing a modified version of the Effective Public Health Practice Project’s quality assessment tool [46]. The evaluation tool, comprised of 19 questions, examined eight key areas of study quality: (a) design, (b) representation, (c) representativeness – selection bias, (d) representativeness – withdrawals and dropouts, (e) confounding factors, (f) data collection, (g) analysis, and (h) reporting (Table 1, Ref. [46, 47, 48]). Using the methodology outlined in McMullan *et al*. [47], studies were assigned 4 to 8 component ratings based on eight criteria, which then determined the overall quality (strong, moderate or weak). For example, the decision rules for a study with six component ratings were: strong (no weak, at least 3 strong), moderate (one weak, less than 3 strong), or weak (two or more weak).

**Table 1.** **Explanation of the scale for assessing the quality of the studies**.

Item	Explanation
Study design	Study design indicated the methodology employed, encompassing experimental, observational, cross-sectional, or longitudinal.
Representativeness	Representativeness assesses how well the study sample reflects the target population.
Representativeness II	Representativeness II concerns the representativeness of the remaining sample after accounting for participant losses.
Confounding factors	Confounding addresses how well the study controlled for or accounted for relevant confounding variables in the sample’s analysis.
Data collection	Data collection evaluates the use of measurement tools with sufficient psychometric properties.
Data analysis	Data analysis examines the analytical methods, comparing basic descriptive techniques to more complex analysis.
Data reporting	Data reporting evaluates the authors’ precision in reporting hypotheses and statistical probability.
Overall score	It’s the average of the prior ratings.

*Note.* Information extracted and expanded from Wermelinger Ávila *et al*., [46, 47, 48].

## Results

The results of the included studies in this systematic review are shown in Table 2 (Ref. [10, 12, 20, 30, 49, 50, 51, 52, 53, 54, 55, 56, 57, 58, 59, 60, 61, 62, 63, 64, 65, 66, 67, 68, 69, 70, 71, 72, 73, 74, 75, 76, 77, 78, 79, 80, 81, 82, 83, 84, 85, 86, 87, 88, 89, 90, 91, 92, 93, 94, 95, 96]).

**Table 2.** **Characteristics of studies included in the systematic review**.

Author(s)	Country	Design	Participants	Evaluated variables	Main contribution
Taylor *et al*., 2008 [49]	United Kingdom (UK)	Descriptive, non-experimental, cross-sectional, comparative	20 participants (12 men and 8 women) aged from 19–60 years old (*M* = 34) with a diagnosis on autism spectrum disorder (ASD), an intelligence quotient (IQ) above 70 and fluent spoken English. The age of autism diagnosis varied widely, from childhood to adulthood (4 to 58 years old); the average time since the diagnosis was 6 years old, with a range up to 16 years.	- Sociodemographic variables: age, gender, age at which they received an autism spectrum diagnosis and fluent spoken English.	- Individuals with ASD are often found to have a higher prevalence of insecure-dismissing attachment compared to individuals without ASD.
			From the 20 participants, 15 had been given a diagnosis of Asperger’s syndrome (AS), four other of autism or high-functioning autism, and one of atypical autism.	- Psychological variables: childhood relationships with parents and other attachment figures (AAI) [50], ASD (Autism Diagnostic Observation Schedule, ADOS) [51], IQ (Weschler Abbreviated Scale of Intelligence, WASI) [52], theory of mind (“reading the mind in the eyes” and “strange stories”), [53, 54].	- Only a few participants had a secure attachment (3 out of 20), which is less common. This could be due to participants’ autism per se or other mental health condition or mood disorders.
			The control group is also formed by 20 participants aged 20–50 (*M* = 34), selected from 39 control transcripts from Adult Attachment Interview (AAI), differentiating two groups: parents whose children were being studied for emotional growth in their community, and adults with mood disorders who were being followed after receiving child psychiatric care.		- Participants’ speech patterns, such as displays of anger or memory lapses, seems to influence the AAI, suggesting these are general speaking traits rather than indicators of attachment-related states of mind.
			In total, twelve individuals with ASD and thirteen individuals in the control group were experiencing mood disorders during their respective AAI.		- Participants’ speech patterns changed during the interviews, especially in how well they communicated, which is a key sign of secure attachment.
Yokotani, 2011 [55]	Japan	Descriptive, non-experimental, cross-sectional, comparative	21 Japanese people with high functional autistic spectrum disorders (HFASD) who attend specialized day-care centers in Sendai, Japan. All participants have at least an IQ of 69 or a 68 verbal IQ. The mean age was 26.66 (*SD* = 8). The participant group was predominantly male, with about four males for each female.	- Sociodemographic variables: age, gender, how many jobs lasting under one month they had.	- The avoidant attachment style alone impacts the job adaptation of people with HFASD in a competitive setting.
				- Psychological variables: autistic tendencies (Autism Spectrum Disorder Quotient, AQ) [56], long-term support (Perceived Emotional Support Network Scales in a family) [57] and avoidant attachment style (Internal Working Model Scale) [58].	- The findings suggested that the avoidant attachment style was an index of the better job adaptation of people with HFASD in a competitive setting. Support persons surrounding them should neither regard their avoidant style as worse adaptation nor encourage them to engage in approach coping with their problems.
					- The present findings also implied the importance of interpersonal cognition of people with HFASD as their avoidant cognition alone impacted their duration of employment.
Lau and Peterson, 2011 [59]	Australia	Descriptive, non-experimental, cross-sectional, comparative	The study included 157 Australian men and women aged between 29 and 71 years. Participants were divided into two main groups. The first group consisted of 75 individuals in a non-clinical control group, who had no family members diagnosed with ASD or any other clinical condition. The second group comprised 82 participants, all of whom had at least one child with a confirmed diagnosis of Asperger Syndrome (AS).	- Sociodemographic variables: age, gender, diagnosis of ASD, spouse or any family members with an ASD diagnosis, child with an ASD diagnosis.	- Married parents with AS in Group 1 predominantly identified with an avoidant attachment style, with nearly 75.00% reporting this pattern, which was significantly more prevalent than in all other groups combined.
			Within this clinical group, three subgroups were identified. The first subgroup, which was of primary interest, included 22 adults who had received a confirmed clinical diagnosis of AS and were also parents of a child with AS. The second subgroup consisted of 11 adults who did not have AS but whose spouse and at least one child were diagnosed with the condition. In the third group, 49 parents had children with AS, while no other adult family members were diagnosed with AS.	- Psychological variables: attachment style (Adult Attachment Style, AAS) [60], marital satisfaction (Quality Marriage Index) [61], parenthood satisfaction (Parenting Sense of Competence) [62].	- In the other three groups, the majority of participants (72.00%) identified with a secure romantic attachment classification.
			The control group (Group 4), consisting of individuals without clinical diagnoses, was carefully matched to the three clinical groups based on age, gender and family size.		- The anxious-ambivalent attachment style was highly uncommon among all groups examined in this research.
					- Means for Groups 1 through 4 did not differ significantly from one another regarding the marital quality scores, suggesting that marital satisfaction was not influenced by whether or not someone in the family had an AS diagnosis.
					- Regarding the parenthood satisfaction, parents of children without AS in the control group showed greater satisfaction with parenthood than parents of children with AS in Groups 1–3. The satisfaction levels within the AS groups were comparable, suggesting the child’s diagnosis was the primary influence.
					- While avoidant attachment is an insecure style, it is also important to remember that this style is not only found in people with AS, but in many adults.
					- The prevalence of secure attachment in adults with ASD is low, at only 15.00%.
Lamport and Turner, 2014 [20]	United States of America (USA)	Descriptive, non-experimental, cross-sectional	249 undergraduate students (122 men and 123 women, 4 individuals declined to specify their gender) aged 18–42 (*M* = 20.35; *SD* = 3.45). The study recruited participants from introductory psychology classes at the college level, offering course credit for their participation. Their average age at which they had their first relationship was 16.10 years (*SD* = 1.90).	- Sociodemographic variables: age, first relationship and origin.	- Individuals with broad autism phenotype (BAP) exhibited higher levels of both attachment anxiety and avoidance.
				- Psychological variables: Broad Autism Phenotype (Broad Autism Phenotype Questionnaire, BAPQ) [63], adult romantic attachment (Experiences in Close Relationships-Revised, ECR-R) [64] and empathy (Empathy Quotient-Short) [65].	- Those with high BAP scores are less prone to having secure romantic attachments. This suggests that romantic relationships could be different for people with BAP compared to those without BAP.
					- The relationship between BAP and attachment avoidance was explained, in part, by empathy.
					- Low empathy levels, associated with high BAP, contribute to higher levels of attachment avoidance.
					- A perceived inability to recognize and respond to emotions, typical of individuals with low empathy, might foster an avoidant attachment pattern.
Gallitto and Leth-Steensen, 2015 [66]	Canada	Descriptive, non-experimental, cross-sectional	326 participants with autism (243 females and 83 males) students of introductory psychology who completed an online questionnaire set for course credit. Their ages ranged between 17 and 33 years old (*M* = 20.80, *SD* = 3.50).	- Sociodemographic variables: age, gender, relationship status.	- The control variables significantly influenced the variation in attachment anxiety with motivational processes, neuroticism and openness emerging as a significant predictor.
				- Psychological variables: adult attachment style (ECR) [67], ASD (AQ) [68], personality characteristics (Personality Mini-Markers) [68], motivational processes (BIS/BAS scales) [69].	- Once the influence of other factors was considered, the connection between ASD and attachment anxiety was no longer significant.
					- The control variables significantly influenced the variation in attachment avoidance with gender, relationship status, extroversion, conscientiousness and agreeableness emerging as significant predictors.
					- After adjusting for several covariates, the significant association between ASD and attachment avoidance persisted.
					- The observation that autism is specifically related to attachment avoidance, even when controlling for other variables, indicates that individuals who score higher ASD traits may find it difficult to form emotionally close romantic connections.
					- Both emotional avoidance and communication issues are frequently observed as features of the BAP.
					- The evidence from this study implies that anxious attachment is not solely determined by BAP traits.
Brandaro and Kroese, 2019 [10]	United Kingdom (UK)	Quantitative, non-experimental, longitudinal (two weeks)	College students, individuals from support groups and online users were asked to participate in the study. They were given a £10 voucher for a popular store and were compensated for any travel costs. A total of 28 participants aged 18–59 (*M* = 35) were recruited.	- Sociodemographic variables: gender, age, previous teaching and siblings.	- Participants’ understanding of attachment showed significant improvement through the study, showing an increased understanding of attachment.
			Participants were not required to submit formal diagnosis of AS, but they all declared that they had received and identify with the diagnosis.	- Psychological variables: intellectual functioning (Weschler Abbreviated Scale of Intelligence, WASI-II) [70], ASD (Autism Spectrum Quotient 10-item, AQ-10) [67], trait emotional intelligence (Trait Emotional Intelligence Questionnaire – Short Form) [71], attachment (Attachment Questionnaire) and DVD adapted by Pearson [72] from ‘Attachment in Practice’ [73].	- There was not significant correlation between emotional intelligence and pre-intervention Attachment Questionnaire scores.
					- There is no significant correlation between intellectual functioning and pre-intervention Attachment Questionnaire improvement scores.
					- Studies demonstrate that a training DVD can effectively teach early attachment behaviors, with lasting knowledge. While some learning occurred before the intervention, it wasn’t significant, which rules out of time or task familiarity as factors. The DVD training clearly improved attachment knowledge.
					- Neither self-reported emotional intelligence nor ASD traits were related to initial knowledge of early attachment behaviors, nor did they predict how much knowledge improved after the intervention.
					- Neither high emotional intelligence nor ASD symptoms were associated with increased knowledge of attachment behaviors before the intervention.
					- A teaching of DVD can effectively improve the limited knowledge of positive attachment behaviors commonly found in adults with AS.
					- Emotional intelligence, IQ and ASD traits did not predict the degree of improvement in knowledge.
Hirokawa *et al*., 2019 [74]	Japan	Descriptive, non-experimental, longitudinal (2011–2014)	Participants consisted of 87,369 pregnant women in Japan, enrolled in the government-funded Environment and Children’s Study (JECS) birth control study. They were recruited during early pregnancy at medical facilities or government offices between January 2011 and March 2014.	- Sociodemographic variables: age, educational attainment, living with other family members and infants’ sex.	- As ASD scores increased, the likelihood of insecure maternal attachment also progressively increased, even after accounting for age, postpartum depression, and other relevant factors.
			The present study is based on the jecs-ag-20, 160, 424 datasets, which includes data from enrollment during the first trimester of pregnancy, during the second or third trimester, at delivery, and when infants were one moth old.	- Clinical variables: postpartum depression, spousal abuse experiences, alcohol consumption, physical activity, first child status and infants’ physical anomalies.	- The ORs for maternal attachment showed a gradual increase as ASD scores increase.
				- Psychological variables: ASD (the Japanese version of the Autism Spectrum Quotient 10-items, AQ-J-10) [75], maternal attachment (Japanese version of the Mother to Infant Bonding Scale, MIBS) [76] and pregnancy and postpartum depression (Edinburgh Postnatal Depression Scale) [77].	- The was no significant link between average social skills and imagination scores and the likelihood of insecure maternal attachment.
					- Although postpartum depression lessened the strength of the ASD-maternal attachment relationship, the connection was still statistically meaningful.
					- Elevated maternal BAP during the second or third trimester of pregnancy was predictor of increased postpartum depression and insecure maternal attachment when infants reached on month of age.
					- Deficits in social skills, according to the AQ-J-10, were linked to four aspects of insecure maternal attachment, excluding feelings of not loving the baby.
					- The relationship between mother’s BAP and maternal attachment was partially influenced by postpartum depression.
					- Postpartum depression amplified the connection between poor imagination and insecure maternal attachment.
					- Postpartum depression, combined with limited imagination, can increase the severity of insecure maternal attachment.
					- Social ability is an important factor in predicting insecure maternal attachment.
					- Maternal BAP levels indicated a likelihood of insecure maternal attachment to the infant.
Beffel *et al*., 2021 [30]	United States of America (USA)	Descriptive, non-experimental, cross-sectional	The study included 263 university students, aged 18 to 25, who were involved in a romantic relationship and had BAP. The majority (79.50%) of the participants were female (*M* = 20.15, *SD* = 1.52), and they were recruited from a university subject pool and received course credit as a compensation.	- Sociodemographic variables: age, gender, race/ethnicity, parent relationships, sibling relationships and romantic relationships.	- Aloofness, pragmatic language and rigidity were positively associated with anxiety attachment.
				- Psychological variables: BAP (BAPQ) [63], relationship satisfaction (Couple Satisfaction Index-16) [78] and attachment styles (ECR-R) [64].	- Avoidant attachment was linked to aloofness, but not to pragmatic language skills.
					- Anxiety attachment and avoidance had negative direct associations with relationship satisfaction.
					- Increased aloofness correlated with both greater anxiety and avoidance in relationships, which was linked to reduced relationship satisfaction.
					- A greater degree of pragmatic language was linked to increased avoidant attachment, which then predicted lower satisfaction in relationships.
					- Higher levels of pragmatic language difficulties were associated with more relationship satisfaction.
					- Increased rigidity correlated with greater anxiety attachment, which led to reduced relationship satisfaction, and conversely, it was linked to lower levels of avoidance.
					- Individuals with BAP traits tend to show more insecure attachment, and this insecurity is linked to reduced satisfaction in their relationships.
Kulasinghe *et al*., 2022 [79]	Australia	Cross-sectional online survey, longitudinal (2019–2020)	Participants were required to meet the following criteria: they must be adults (+18), mothers of children with ASD (under 10 years), Australian residents, and English-literate.	- Sociodemographic variables: mother age, marital status, maternal status, parent education level, origin, child age, child age at diagnosis, child gender and financial hardship in past 12 months.	- Higher scores for BAP related to higher scores to both avoidance and anxiety attachment.
			The final sample is formed by 231 participants. The mean ages are: 39.19 for mothers (*SD* = 5.30), 6.68 for children (*SD* = 2.14), and 4.24 for child age at diagnosis (*SD* = 1.92).	- Clinical variables: child diagnosis, child comorbidities and interventions accessed.	- Parents who had positive perceptions of their parenting were more likely to have emotionally available relationships with their children.
				- Psychological variables: BAP (BAPQ) [63], attachment style (ECR-R) [64], maternal mental health (DASS-21) [80], psychological inflexibility (AAQ-II) [81], parenting experience and emotional availability (Emotional Availability Self Report) [82], autism family experience (Autism Family Experience Questionnaire, AFEQ) [83].	- The presence of maternal stress and anxiety negatively impacted mutual attunement. This could be explained by stress-induced oversights of child initiations or anxiety-driven preoccupation, both hindering attunement.
					- It was found that affect quality is influenced by both parents and child factors. More precisely, maternal anxiety only predicted poorer affect quality among mother with high BAP, not among those with low BAP.
					- Compared to those with lower BAP scores, those with higher scored indicated reduced mutual attunement and increased psychological inflexibility, avoidant attachment, depressive symptoms, anxiety, and stress.
Lee *et al*., 2022 [12]	Australia	Cross-sectional online survey	Participants were 126 Australian adults with ASD (*M* = 32.68 years, *SD* = 10.10). A majority of the participants were female (55.60, *n* = 70), and a significant portion identified as transgender (19.80%, *n* = 25). Participants were self-identified with autism, being the most frequently endorsed diagnosis AS (*n* = 55), followed by ASD (*n* = 21), and autism (*n* = 20).	- Sociodemographic variables: age, origin, biological sex, transgender identity, relationship status, current living situation, annual income in past year, highest qualification attained, employment and studying or not.	- Most participants (*n* = 111, 88.10%) listed their biological mother as their main caregiver, and very few mothers (*n* = 2, 1.80%) had a diagnosis of ASD. Of the participants who named their biological father as their primary caregiver (*n* = 12, 9.50%), only a small portion (*n* = 3, 25.00%) reported their father had an ASD diagnosis.
				- Clinical variables: diagnoses, concurrent neurodevelopmental diagnoses, specific learning disorder (SLD), parental diagnosis and other concurrent diagnoses.	- A significant portion of secondary caregivers were biological fathers (*n* = 100, 79.40%), with only 4.00% diagnosed with ASD. In the group where biological mothers were secondary caregivers, 22.20% (*n* = 2) reported a perceived presence of ASD.
				- Psychological variables: autistic traits (BAPQ) [63], parental diagnosis (ASD *yes/no*), childhood experiences of parental care and overprotection (The Parental Bonding Instrument) [84], current adult attachment style (ECR-R) [64], psychological inflexibility (AAQ-II) [81], social engagement (The Lubben Social Network Scale-6) [85], mental health (DASS-21) [80].	- Anxious attachment was linked to increased depression, anxiety and stress, but avoidant attachment was only associated with increased depression.
					- Psychological inflexibility and anxious attachment positively correlated with depression and anxiety, whereas avoidant attachment showed a negative correlation with anxiety and stress.
					- Among participants with both primary and secondary caregivers, multiple regression analysis showed that increased psychological inflexibility, anxious attachment and reduced vocational engagement were associated with higher depression scores.
					- Psychological inflexibility, anxious attachment, and secondary caregiver overprotectiveness positively correlated with anxiety, while avoidant attachment showed a negative correlation with both anxiety and stress.
					- The relationship between primary parental care and depression and anxiety was entirely explained by attachment anxiety, which did not explain any other relationship examined.
					- Attachment avoidance play no mediating role in the relationships examined in this study.
					- Both higher care and lower overprotection correlated with fewer depressive symptoms, but only lower overprotection correlated with reduced anxiety.
					- Results indicated that attachment anxiety completely explained the relationship between primary parental care and depression and anxiety, but attachment avoidance had no mediating effect on any of the relationships studied.
Murray *et al*., 2022 [86]	Ireland and China	Descriptive, non-experimental, cross-sectional, comparative	Participants included 230 adults with AS (*M* = 31.32, *SD* = 11.03) and 272 adults in a control groups (*M* = 29.51, *SD* = 13.53). The control group was assembled via social media, posters, and a university student participant system, where students received course credit.	- Sociodemographic variables: age, country region.	- A significantly larger portion of the ASD group (9.10%) exhibited gaming disorder symptoms above the clinical threshold compared to the typically developing group (2.90%). Additionally, the ASD group reported a higher mean level of gaming disorder symptoms (*M* = 0.28, *SD* = 0.29) than the typically developing group (*M* = 0.17, *SD* = 0.25).
			To ensure the control group’s accuracy, 23 individuals were removed due to scoring above the ASD cutoff on the AQ-10 or being in the diagnostic process. Additionally, two participants in the ASD group who self-reported their diagnosis were excluded.	- Psychological variables: ASD (AQ-10) [87], GD (Ten-Items Internet Gaming Disorder Test) [88], social functioning (Social Functioning Questionnaire) [89], extraversion (NEO Five-Factor Inventory-3) [90], emotional regulation (ERQ) [91], peer attachment (Inventory of Parent and Peer Attachment) [92] and gelotophobia symptomatology (The Geloph <15>) [93].	- The study used hierarchical regression to explore which factors predict gaming disorder, using extraversion, peer attachment, emotional regulation, and social functioning as independent variables. The results demonstrated that peer attachment was a significant predictor, explaining 5.00% of the variance.
					- Gaming disorder was predicted by social functioning, extraversion, emotional regulation, and peer attachment.
					- Of the measured factors, only cognitive reappraisal (within emotional regulation) and alienation (within peer attachment) significantly predicted the outcome.
Fukui *et al*., 2023 [94]	Japan	Descriptive, non-experimental, cross-sectional, comparative	2692 postpartum women (*M* = 31.60 years; *SD* = 4.80) who visited the participating obstetric institutions at 1 moth postpartum. The sample was divided into three groups according to the AQ scores: normal, BAP and medium autism phenotype (MAP).	- Sociodemographic variables: age.	- In the normal, BAP and MAP groups, higher levels of ASD were significantly related to higher mean scores in emotional distress, anxiety, depression and maternal-infant bonding.
				- Clinical variables: primiparous/multiparous, natural conception/others, full term/preterm delivery and vaginal delivery/caesarean section.	- Higher scores for anger and rejection were related to higher scores in social skills, attention switching, communication and imagination in the autism subscale and lower scores in attention to detail.
				- Psychological variables: autistic traits (AQ) [56], depression and anxiety (Hospital Anxiety and Depression Scale) [95, 96] and maternal-infant bonding in the early postpartum period (MIBS) [76].	- Women in the perinatal period who have poor social skills but are detail-oriented may develop better face recognition by focusing more on their infants’ eyes, which could lead to stronger mother-infant bonding.
					- Greater attentiveness towards the child can positively influence bonding.
					- While maternal ASD traits showed a moderate link with anxiety and depression, their relationship with maternal-infant bonding at one month after birth as minimal.

### Characteristics of the Studies: Sample and Design

The sample consists of 92,213 participants, 91,078 (98.99%) women and 346 (0.38%) men. Only two studies did not specify the gender of the sample [30, 86], one study did not specify the gender of the control group [49] and four participants form another study declined to specify their gender [20], leaving a total of 789 (0.85%) participants with the gender not specified. The sample ages ranged from 19 to 71 years, with a mean age of 29.73 years, and one study did not specify [59] (Table 3).

**Table 3.** **Descriptive statistics of the sample**.

Variable assessed		N
Biological sex		
	Male	346
	Female	91,078
	Not specify	789
Studies that comprised children with ASD		2
Studies of parents with BAP		4
Studies with adults with BAP in a romantic relationship		3
Studies taking into account other variables		5
Study design		
	Longitudinal	3
	Cross-sectional	9
Studies with an official ASD diagnosis		4
Studies with no official ASD diagnosis		4
Studies that do not specify if the diagnosis is or not official		4
Studies with inclusion and/or exclusion criteria		8
Studies conducted in the last decade		8
Country/state where studies were conducted		
	Japan	3
	Australia	3
	USA	3
	UK	2
	Both UK and China	1
Studies who offered a compensation for participation		5

*Notes. N* = population/sample size.

A total of twelve studies were included in the sample, of which 33.33% (*n* = 4) recruited parents with BAP [59, 74, 79, 94] and two of them also comprised their children with ASD [59, 79], 25.00% (*n* = 3) recruited adults with ASD who were in a romantic relationship [20, 30, 66], 8.33% (*n* = 1) recruited adults with ASD to study their attachment style regarding their ASD [49], 33.33 % (*n* = 4) recruited adults with ASD to study their attachment style taking into consideration other variables [10, 12, 55, 86] (Table 3). Only two of the studies explicitly mention whether the children of parents with ASD or autistic traits also have an ASD diagnosis [59, 79]. In the first study [59], one of the groups analyzed consisted of 49 parents with a child diagnosed with Asperger’s syndrome. The second study [79] included, as part of its inclusion criteria, mothers of children under 10 years old diagnosed with ASD. The remaining studies focused exclusively on the adult population and did not report whether participants had children with ASD.

In terms of research design, only three studies of the twelve (25.00%) were longitudinal; the first one took mothers measures in four points in time (during first the second or third trimester of pregnancy, childbirth, and the baby’s first month of life), during a recruiting time from 2011 to 2014 [74], the second one took place over two weeks [10], and the third one collected data from October 2019 to January 2020 [79]. The remaining studies were cross-sectional [12, 20, 30, 49, 55, 59, 66, 86, 94] (Table 3).

Regarding the ASD or BAP diagnosis, only 33.33% (*n* = 4) of the studies included adults with an official ASD diagnosis [49, 55, 59], 33.33% (*n* =4) of the studies comprised adults with no official ASD or BAP diagnostic [10, 12, 30, 94] and 33.33% (*n* = 4) does not specify if the diagnosis were official or not [20, 66, 74, 79] (Table 3).

Regarding the criteria for inclusion or exclusion of papers, participants of all studies were over 18 years. 33.33% studies (*n* = 4) did not report any explicit inclusion or exclusion criteria [10, 30, 65, 86], 58.33% studies (*n* = 7) did report explicit inclusion and exclusion criteria [12, 20, 49, 55, 59, 74, 94] and only 8.33% studies (*n* = 1) did report inclusion but not exclusion criteria [79]. Furthermore, only one of the twelve studies reports that the sample presents other disorders: while mood disorders were most common in the control group, depression, anxiety, schizoaffective disorder, ADHD and dyslexia were the most frequent in the ASD sample [49] (Table 3).

The studies spanned 15 years, from 2008 to 2023. A significant proportion of the studies, 66.67% (*n* = 8), were conducted within the last decade, reflecting a heightened interest in attachment and adults with ASD [10, 12, 30, 66, 74, 79, 86, 94]. 25.00% of the studies were conducted in Japan [55, 74, 94], 25.00% in Australia [12, 59, 79], 25.00% in the USA [20, 30, 66], 16.67% in the UK [10, 49] and only one study (8.33%) was conducted both in the UK and China [86]. Additionally, only four studies (33.33%) specify the race/ethnicity or country region of the participants [20, 30, 49, 86] (Table 3).

Regarding the compensation for being part of the sample, five of the twelve studies (41.67%) rewarded participants for their participation on the study with course credit [20, 30, 66, 86] and a £10 high street voucher as well as payment for any travel expenses [10] (Table 3).

### Variables Assessed

#### Attachment and Autism

In terms of attachment variables, assessments included adult attachment and maternal bonding to their children. The scales that were used to assess adult attachment were: Experiences in Close Relationships (ECR) [12, 20, 30, 64, 66, 67, 79], Adult Attachment Interview (AAI) [49, 50], Adult Attachment Style (AAS) [59, 60], Attachment Questionnaire [10, 72], Internal Working Model Scale [55, 58], and Inventory of Parent and Peer Attachment [86, 92]. Only four of the five studies that used the ECR scale employed it in the revised version [12, 20, 30, 64, 79]. On the other hand, the scales used to assess maternal bonding were the Mother to Infant Bonding Scale (MIBS) [74, 76, 94] Japanese version, and the Parental Bonding Instrument [12, 84].

Regarding ASD or autistic traits assessment, studies used the following scales to measure those traits: 50.00% used the Autism Spectrum Disorder Quotient (AQ) [10, 55, 56, 66, 67, 74, 75, 86, 87, 94], 33.33% used the Broad Autism Phenotype Questionnaire (BAPQ) [12, 20, 30, 63, 79], 8.33% used the Autism Diagnostic Observation Schedule (ADOS) [49, 51] and 8.33% used a parental diagnosis by ASC *yes/no* [12]. Only one study did not used any measure to assess ASD in participants [59]. From the six studies that used the AQ, it was employed with both normal [55, 56] and 10-item version [10, 66, 67, 74, 75, 86, 87, 94], and Hirokawa *et al*. [74, 75] used the Japanese version.

#### Other Variables

Regarding psychopathology, other variables were taken into consideration. 16.66% of the studies (*n* = 2) assessed the intelligence quotient using the Weschler Abbreviated Scale of Intelligence (WASI) in both first [49, 52] and second version [10, 70]. Mental health in both mothers and general population were measured in 33.33% studies (*n* = 4) by the Edinburg Postnatal Depression Scale [74, 77], Depression, Anxiety and Stress Scale (DASS-21) [12, 79, 80] and the Hospital Anxiety and Depression Scale [94, 95, 96].

There were also considered the marital satisfaction by the Quality Marriage Index [59, 61] and the relationship satisfaction using the Couple Satisfaction Index – 16 [30, 78], as well as parenthood satisfaction, parenting experience and emotional availability using the Parenting Sense of Competence measure [59, 62] and the Emotional Availability Self-Report [79, 82]. Emotional support, emotional intelligence and emotional regulation were also measured in 25.00% of the studies by the Perceived Emotional Support Network Scales in family [55, 57], the Trait Emotional Intelligence Questionnaire-Short Form [10, 71] and Emotional Regulation Questionnaire (ERQ) [87, 91].

Besides ASD and BAP measures, there were measured other ASD difficulties such as theory of mind by “reading the mind in the eyes” and “strange stories” [49, 53, 54], psychological inflexibility by the Acceptance and Action Questionnaire-II [12, 79, 81], and social engagement by the Lubben Social Network Scale-6 [12, 85]. Other variables were also assessed using different measures: the Empathy Quotient-Short was employed to measure the empathy [20, 65]; personality characteristics were measured by Personality Mini-Markers [66, 68]; BIS/BAS scales were used to assess motivational processes [66, 69]; gaming disorder was measured by the Ten-Items Internet Gaming Disorder Test [86, 88]; gelotophobia was measure by The Geloph <15> [86, 95], and the NEO Five-Factor Inventory-3 was used to assess extraversion [86, 90].

#### Main Results of Attachment in Adults With ASD Related to Other Psychological Variables

It has been shown that adults with an ASD diagnosis are more likely to have an insecure/dismissing attachment style compared to the non-clinical population [49] and show lower scores for secure attachment style [49, 59]. This could be due to participants’ autism per se or other mental health condition or mood disorders, as it has been seen that mood disorder could be associated with insecure and disorganized attachment [49]. Although insecure attachment style is common in adults with ASD, it is important to remember that this style is not only found in people with AS, but in many adults [59].

Also, higher scores on BAP are associated with higher levels of both attachment anxiety and avoidance [20, 79]. Regarding romantic relationships, individuals who scored high on BAP report less secure romantic attachments [20] and lower relationship satisfaction [30]. Beffel *et al*. [30] have shown that higher levels of aloofness, pragmatic language and rigidity are associated with lower relationship satisfaction as these traits are related to higher anxiety and avoidance, suggesting that they could be less able or willing to experience emotional closeness with romantic partners [66].

Additionally, it has been observed that adults with AS have a reduced awareness of positive attachment behaviors, which can be significantly improved through the use of instructional DVDs [10]. Besides, attachment style could also impact job adaptation of people with high functioning autism spectrum disorder (HFASD), as having an avoidant attachment style could be an index of a better job adaptation [55]. Furthermore, peer attachment along with social functioning, extraversion and emotional regulation predicted gaming disorder. Murray *et al*. [86] showed that participants with ASD reported higher rates of gaming disorder symptoms than typically developing participants.

On the other hand, and regarding the studies with parents and mothers with ASD or BAP [59, 74, 79, 94], it has been shown that almost three quarts of the married parents with ASD have higher scores in the avoidant attachment style than general population; nevertheless, the marriage was equally satisfying to respondents regardless of their diagnosis. However, in terms of parenthood satisfaction, the general population showed more satisfaction from parenthood than individuals with AS child or themselves [59].

As for other psychological variables related to mothers’ BAP, it is observed that these traits during the second or third trimester of pregnancy were associated with a greater likelihood of postpartum depression and insecure maternal attachment when infants were aged one month old [74], although Fukui *et al*. [94] reported that maternal BAP is weakly related to maternal-infant bonding at one month postpartum. Mothers with higher BAP scores experience lower levels of mutual attunement and higher psychological inflexibility, attachment avoidance, depressive symptoms, anxiety and stress than those with lower BAP scores [79]. Also, social ability and mothers’ BAP are an important factor in predicting insecure maternal attachment [74]. The emotional connection between parent and child is influenced by various factors in both, and greater parental focus on the child can be a valuable tool for enhancing bonding [79, 94].

Lastly, regarding attachment and its relationship with other variables, Lee *et al*. [12] observed that higher anxious attachment is related to greater depression, anxiety and stress whereas higher adult avoidant attachment was associated with higher depression and lower anxiety and stress. Similarly, higher scores for psychological inflexibility, anxious attachment and secondary caregiver overprotectiveness predicted lower scores for anxiety and stress. Equally, it has been reported that the link between the primary care and depression and anxiety if fully explained by attachment anxiety. Increased parental care and reduced overprotection were associated with fewer depressive symptoms, while only reduced overprotection was associated with fewer anxiety symptoms.

On the other side, empathy mediates the relationship between BAP and attachment avoidance as higher BAP is associated with higher attachment avoidance through its association with low empathy [20]. At last, Gallitto and Leth-Steensen [66] reported that motivational processes, neuroticism and openness are predictors of attachment anxiety, and gender, relationship status, extroversion, conscientiousness and agreeableness emerge as attachment avoidance predictors.

#### Quality Assessment

Using PRISMA guidelines [42], A.S evaluated the articles and assigned quality ratings from 1 to 5 (most likely to be biased or lowest quality). As shown in Table 4 (Ref. [10, 12, 20, 30, 49, 55, 59, 66, 74, 79, 86, 94]), the average quality score was 2.13, reflecting a moderate quality level across the reviewed studies.

**Table 4.** **Article quality assessment**.

Author(s)	Study design	Representation	Representation II	Confounding factors	Data collection	Data analysis	Data reporting	Overal rating
Taylor *et al*. [49]	3	3	N/A (do not follow up)	1	1	2	1	Moderate
Lau and Peterson [59]	3	2	N/A (do not follow up)	3	1	2	1	Moderate
Yokotani [55]	4	3	N/A (do not follow up)	1	2	4	1	Moderate
Lamport and Turner [20]	4	4	N/A (do not follow up)	4	1	2	1	Moderate
Gallitto and Leth-Steensen [66]	4	4	N/A (do not follow up)	3	1	2	2	Moderate
Brandaro and Kroese [10]	1	3	1	3	1	2	2	Moderate
Hirokawa *et al*. [74]	1	2	1	1	1	2	1	Strong
Beffel *et al*. [30]	4	4	N/A (do not follow up)	4	1	2	2	Moderate
Kulasinghe *et al*. [79]	1	2	1	1	1	2	1	Strong
Lee *et al*. [12]	3	3	N/A (do not follow up)	1	1	3	1	Moderate
Murray *et al*. [86]	3	3	N/A (do not follow up)	4	3	2	2	Moderate
Fukui *et al*. [94]	3	2	N/A (do not follow up)	1	1	3	1	Moderate

## Discussion

The aim of the present systematic review was to determine which attachment style is the most common in adults with ASD. Additionally, it sought to understand the relationship between the attachment styles in adults with ASD and other psychological, relational and clinical variables. After an exhaustive literature review following the PRISMA guidelines [42], two independent reviewers assessed the studies. Finally, this review analyzed data from 12 studies involving 91,078 women and 346 men. Key findings emphasize that adults with ASD are more likely than general population to develop an insecure attachment, as well as having higher scores at anxiety and avoidant attachment. Besides, parents exhibiting higher levels of BAP along with other disorders may demonstrate difficulties in establishing secure attachments with their infants.

The literature consistently indicates that insecure attachment is the most commonly identified attachment style in adults with ASD or BAP [20, 49, 59, 79], showing lower levels of secure attachment. This could be attributed to the ASD traits themselves or to comorbid disorders such as depression, anxiety, and/or stress [12, 49, 59, 66]. Factors like personality traits, gender and relationship status also play a role [66].

This systematic review indicates that attachment styles have also a great impact in relationship satisfaction, job adaptation and gaming disorder [20, 30, 55, 66, 79, 86]. Regarding the relationship satisfaction, individuals with ASD or BAP tend to exhibit anxious and avoidant attachment styles, which are associated with lower relationship satisfaction and less emotional closeness in romantic partnerships [20, 30, 66, 79], consistent with prior research [97, 98, 99].

Additionally, although adults with ASD struggle with attachment understanding due to social communication difficulties, psychological interventions can improve knowledge of early attachment behaviors, with effects lasting at least a week [66]. These findings are consistent with Pearson *et al*.’s [100]; however, they show mixed results: while one study found improvement lasting a week [66], another found knowledge did not endure after two weeks [100], possibly due to the longer follow-up period. Future research should explore how anxiety, depression, attachment styles, and gender influence attachment learning in ASD. There is also a need to develop specific assessment tools to measure attachment understanding and create effective interventions.

On the other hand, other studies highlight the crucial role of the parent-child dyad in families where both the parent and the child exhibit ASD or BAP, emphasizing the significance of both parental and child-specific variables in understanding how the bond is formed. First, although marital satisfaction is similar between ASD individuals and general population, parents with ASD report lower parenthood satisfaction than those without, possibly due to co-occurring mental health conditions or difficulties with theory of mind, impacting parent-child attachment [59, 74, 79, 94]. Furthermore, the literature consistently supports this idea, suggesting that postpartum depression can interfere with the development of a secure attachment between mother and infant, acting as a significant barrier to bonding [101, 102].

Also, parents with BAP often experience challenges such as psychological inflexibility and difficulties in parenting, which can significantly impact their mental health and the ability to establish secure attachment. These challenges, in turn, can disrupt the parent-child relationship, particularly mutual attunement [79]. They also suggest that mothers with higher BAP scores exhibited increased psychological inflexibility, attachment avoidance and poorer mental health, all of which were associated with lower levels of mutual attunement. This aligns with previous research reporting poorer mental health outcomes in individuals with elevated BAP [99].

### Limitations

Despite the strengths of our study, some limitations must be considered when interpreting the results of this systematic review. Most studies exhibit a reliance on non-probability sampling methods, specifically convenience sampling, which may introduce systematic bias into the findings. Moreover, several articles have a small, non-representative sample, with a predominant female gender, which is not representative of ASD theoretically. On the other hand, the studies conducted are cross-sectional and descriptive, which makes it difficult to study the relationship between the different variables in greater depth and to establish casual relationships between them. Incentives, such as monetary or course credit, offered in three studies could also introduce bias and affect the overall findings. To mitigate these limitations, future research should prioritize rigorous methodological approaches, including random sampling techniques and the recruitment of larger sample sizes within a longitudinal research design.

One important limitation of this study is the lack of consideration of ASD symptom severity in the included articles. Although the severity of ASD symptoms plays a crucial role in attachment and can influence the results, none of the studies in this systematic review accounted for this factor. As a result, it is difficult to determine how different levels of symptom severity may affect attachment styles and, consequently, the findings of the present study. Future research should address this limitation by incorporating ASD severity as a variable to better understand its influence on attachment patterns.

Furthermore, a significant limitation of these studies is the heterogeneity in the measurement instruments used, which precludes the possibility of conducting meta-analysis and therefore limits the generalizability of the findings. Additionally, another limitation is the heterogeneity in the variables assessed, which prevents direct comparisons and limits our understanding of the relationship with attachment styles. Regarding ASD assessments, the lack of professional ASD diagnosis in some studies, combined with the reliance on self-reported ASD and BAP diagnosis, as well as self-reported measures for attachment, reduce considerably the confidence with which we can generalize the findings. Additionally, the percentage of woman who have participated in the studies is significantly higher than that of men, which may make it difficult to generalize the results to this population. Future research should consider expanding the male sample to obtain more specific and generalizable results. However, the predominance of female participants is a common phenomenon in research, reflecting a broader trend in study participation.

On the other hand, although there is increasing interest in exploring attachment styles in adults with ASD, the current literature is predominantly focused on child and adolescent samples. This paucity of adult-focused research hinders our ability to establish a comprehensive understanding of the relationship between attachment and ASD in adulthood. Consequently, there is a critical need for additional research to better understand the casual mechanisms underlying these relationships and develop evidence-based interventions designed to foster secure attachment and to mitigate the prevalence of comorbid disorder. Additionally, future studies should address this gap by incorporating emotional aspects into their analyses, allowing for a more comprehensive understanding of how attachment styles interact with emotional processes in this population. Lastly, the lack of information regarding whether participants have children with an ASD diagnosis represents a limitation in the existing literature. Future research should explore this aspect to better understand the potential intergenerational transmission of autistic traits and its implications for family dynamics and support needs.

Finally, the present systematic review’s limitations include the stringent inclusion criteria employed in the database search, resulting in a limited database search, leading to a smaller final number of articles included. Nevertheless, most studies included were conducted within the past the years. Another significant limitation of this review is the lack of a comprehensive search of the references of the included studies. Such a search could have identified additional relevant studies and expanded the scope of the review.

To our knowledge, this is the first systematic review to explore the relationship between attachment styles in adults with ASD and a range of other variables. The systematic search, guided by stringent inclusion criteria, surpasses the limitations of previous article reviews by providing a more comprehensive and rigorous synthesis of the existing literature. In addition, to ensure rigor, two independent evaluators were involved in all stages of the review process, with inter-rater reliability assessed at each step. Future research directions include investigating the psychological factors that impact attachment styles in adults with ASD or BAP. Furthermore, it is essential to determine the most influential variables in the formation of parent-child bonds.

### Practice Implications

This systematic review can contribute to a deeper understanding of the role of attachment styles in the broader context of adults with ASD and their associations with other psychological variables. Interventions can be designed to enhance the understanding of attachment styles within this population and with the aim of improving the quality of the attachment bonds, and even carry out preventive interventions in families with children who have been diagnosed with ASD, promoting secure attachment by working on the factors that hinder the formation of this type of bond. Another strong contribution is the work with adults, highlighting the importance of creating interventions with them despite not having a clinical ASD diagnosis and providing them the necessary resources for optimal attachment development, promoting well-being in adulthood.

Lastly, the results of the present research also allow us to create interventions aimed at improving emotional bonds with the purpose of promoting healthier romantic and family relationships, developing greater satisfaction and well-being in these types of bonds, as well as better results in their parenting, for example. Both parental and child factors are crucial for bonding. While insecure attachment is a common feature in adults with ASD, it is essential to consider the impact of other external factors.

## Conclusion

Based on the results obtained in this systematic review, it can be concluded that adults with ASD or BAP have an insecure attachment style compared to general population, leading to lower relationship satisfaction, higher rates of gaming disorder and poorer mental health. The difficulties they experience with social communication, interaction and imagination make it difficult when understanding attachment, but their knowledge can be improved by psychological interventions. Also, parents with ASD or BAP show lower parenthood satisfaction and may experience challenges in establishing a secure attachment with their children, highlighting the importance of considering both parental and child variables in understanding how this bond develops. This systematic review highlights the importance of developing interventions with adults with ASD with the aim to establish better bonding and reaching greater well-being and mental health.

## Availability of Data and Materials

Not applicable.

## Supplementary Material

Supplementary material associated with this article can be found, in the online version, at https://doi.org/10.62641/aep.v53i4.1928.
